# Laser hemorrhoidoplasty vs. stapled hemorrhoidopexy: a meta-analysis of clinical outcomes with considerations in surgical anatomy

**DOI:** 10.1007/s00423-026-04346-2

**Published:** 2026-11-12

**Authors:** Dimosthenis Chrysikos, Nikolaos Taprantzis, Amir Shihada, Theodoros Mariolis Sapsakos, Dimitrios Filippou, Theodore Troupis

**Affiliations:** Department of Anatomy, Athens Medical School, National and Kapodistrian University of Athens, Mikras Asias 75, Athens, 11527 Greece

**Keywords:** Laser hemorrhoidoplasty, Stapled hemorrhoidopexy, Hemorrhoids, Meta-analysis

## Abstract

**Purpose:**

Hemorrhoidal disease is a common colorectal condition affecting millions. Recently, minimally invasive approaches have gained popularity for their faster recovery. However, current literature lacks a comprehensive comparison between these two methods. This systematic review and meta-analysis compares the efficacy and outcomes of stapled hemorrhoidopexy (SH) and laser hemorrhoidoplasty (LH).

**Materials and methods:**

A systematic search of PubMed, Embase, Web of Science, and Scopus identified studies comparing SH and LH. Patient cohorts were analyzed for recovery, perioperative outcomes, and complications. Meta-analysis was performed using R to calculate pooled Odds Ratios (OR) and Weighted Mean Differences (WMD).

**Results:**

LH demonstrated a significantly shorter mean operative time compared to SH (25.9 vs. 33.0 min; MD = 7.14). The stapled approach was associated with significantly higher rates of postoperative urinary retention (7.7% vs. 3.0%; OR = 1.92), stenosis (4.5% vs. 0%; OR = 9.74), and longer hospital stays (MD = 16.32). No significant differences were observed regarding estimated blood loss (MD = 2.49), return to normal activity, readmission, postoperative bleeding, or overall VAS pain scores at any time point (12 h, 24 h, 1 week, 2 weeks, and 3 months). Subgroup analyses indicated LH yielded significantly lower pain at 3 months in severe cases.

**Conclusion:**

LH and SH showed no statistically significant differences across several reported short-term outcomes, including postoperative pain, bleeding, and time to return to normal activities. While LH was associated with shorter operative times, reduced hospital stays, and lower rates of urinary retention and stenosis in the available studies, these findings must be interpreted with caution. Crucially, the true comparative therapeutic efficacy of these techniques remains uncertain. Because relevant variables like symptom resolution, patient-reported outcomes, disease-specific functional outcomes were insufficiently assessed, while recurrence data are limited by heterogeneous and inadequately reported follow-up, this analysis permits conclusions regarding short-term perioperative recovery, but not robust conclusions regarding comparative long-term success and efficacy.

**Supplementary Information:**

The online version contains supplementary material available at https://doi.org/10.1007/s00423-026-04346-2.

## Introduction

Hemorrhoidal disease is one of the most common anorectal disorders, that affects millions of people worldwide, especially after the 4th decade. Through the technological developments in the recent years, physicians are able to utilize an array of different operative techniques in order to successfully manage hemorrhoidal disease in many patients. Precisely, surgical operation is indicated in any patient with low-grade hemorrhoids that are refractory to any medical/non-surgical management. Furthermore, patients with high grade disease or strangulated hemorrhoids should also undergo surgical operation [1]. Among others, options include open and closed hemorrhoidectomy, harmonic scalpel, LigaSure, laser hemorrhoidoplasty and stapled hemorrhoidopexy [2]. In general, stapled hemorrhoidopexy is considered to be a good surgical option that is associated with less pain, faster recovery, and shorter hospital stay compared to the classic hemorrhoidectomy. Furthermore, the availability of the stapler devices and the increasing expertise in this technique has established its positions as one of the main treatment options. However, studies have shown an alarming rate of postoperative complications following this procedure, an observation that makes many patients reluctant to undergo this operation [3].

On the contrary, laser hemorrhoidoplasty is a more recent minimally invasive option that offers a potentially less morbid, painless, single day procedure that has quickly become popular among patients [4]. Despite the presence of multiple available management options for hemorrhoids, a general consensus regarding the optimal surgical treatment does not exist. Although previous literature has compared the outcomes, morbidity, and safety of laser and stapled techniques to the open traditional approach, no systematic review with a statistical meta-analytic approach exists that directly compares the two minimally invasive approaches.

While conventional excisional hemorrhoidectomy remains highly effective, it is associated with significant postoperative pain and delayed recovery. This drawback has driven the adoption of less invasive alternatives like stapled hemorrhoidopexy and laser hemorrhoidoplasty [4]. These alternatives, however, rely on different anatomical principles to achieve symptom relief. The former is a mechanical, resective procedure where the hemorrhoids are lifted and pexed using a stapler-assisted technique to remove tissue above the dentate line, leading to reduced pain; however, it still carries specific risks such as postoperative bleeding, anal stenosis, and recurrence. Laser hemorrhoidoplasty is a newer, sphincter-saving technique that operates on a thermal principle, where the enlarged hemorrhoidal cushions are ablated by diode laser energy, causing submucosal tissue shrinkage while leaving the overlying mucosa intact [3, 5]. This targeted coagulation aims to further minimize tissue trauma, theoretically resulting in even less postoperative discomfort and a lower complication rate compared to the stapled technique [3, 5].

While proctological guidelines suggest certain indications for these procedures, such as reserving stapled hemorrhoidopexy primarily for grade III disease while targeting laser hemorrhoidoplasty for milder grades, surgical practice frequently blurs these boundaries. Recent literature demonstrates that both techniques can be applied across a mixed spectrum of grades II, III, and IV in an effort to provide patients with minimally invasive alternatives to open surgery [5–7]. Because current clinical practice does not always adhere to strict grade-specific isolation, a direct comparison is important to help identify which minimally invasive modality provides the most favorable balance of reduced operative time, minimized perioperative morbidity, and expedited return to daily activities.

Due to the nature of the available data in the primary literature, this study aims to systematically evaluate the perioperative safety profile, morbidity, and short-term recovery metrics of laser hemorrhoidoplasty versus stapled hemorrhoidopexy, rather than drawing comparative conclusions on long-term therapeutic efficacy.

## Materials and methods

A literature retrieval was executed across four major electronic databases: Scopus, Embase, Web of Science, and PubMed. To capture all relevant cohorts for our analysis, the search strategy utilized various combinations of the following query terms: “stapled hemorrhoidopexy vs laser hemorrhoidoplasty”, “minimally invasive” AND “treatment for hemorrhoids”, “stapler vs laser treatment” AND [“hemorrhoids” OR “hemorrhoidal disease”], “stapled vs laser hemorrhoidoplasty”, “effectiveness of laser vs stapled hemorrhoidal treatment”, “stapled vs laser” AND [“hemorrhoidopexy” OR hemorrhoidoplasty”] AND “outcomes” AND “safety”, in order to collect data that would fit the criteria of our review. The inclusion criteria were strictly limited to English-language publications. To guarantee a comprehensive yield, a secondary manual cross-reference check of the bibliographies from pertinent articles was also performed. Given that our methodology relies exclusively on previously published, publicly accessible data, the requirement for Institutional Review Board clearance and informed patient consent was not needed. PROSPERO registration was also completed: CRD420261458457. The study followed PRISMA guidelines [Supplementary File 1].

The final set of studies and articles was decided based on the predefined selection of criteria, which included: studies that compared the outcomes exclusively between laser and stapled techniques for hemorrhoidal disease. On the other hand, studies that included patients who also underwent a different type of treatment for hemorrhoidal disease, other than the two aforementioned, were not included.

Additionally, due to the nature and the availability of the current data, the collection of outcomes that were decided to be assessed primarily focused on perioperative safety and short-term recovery metrics. These included: operative blood loss (ml), operative time (min), length of stay (hours), return to activity (hours), urine retention, recurrence, stenosis, Visual Analog Scale (VAS) pain at 12 h, 24 h, 1 week, 2 weeks, 1 month and 3 months, bleeding and readmission. While an initial effort was made to report specific symptom-related outcomes (e.g., resolution of prolapse, soiling, or pruritus) to evaluate long-term therapeutic efficacy, the lack of uniform reporting across the primary literature precluded a systematic analysis of these variables. Subgroup analyses were also conducted in order compare the two surgical techniques across different grades of hemorrhoidal disease. The studies were divided based on their inclusion of grade IV disease and thus evaluating the comparison of stapled vs. laser techniques between studies with mild and more severe hemorrhoids. Due to the limited number of studies, a meta regression for this analysis was not possible to be performed.

Finally, this review did not include animal studies, abstracts from conferences or any study that did not report the data sufficiently. Because of the substantial clinical heterogeneity in follow-up duration across the included primary studies, a quantitative meta-analysis for recurrence rates was deemed methodologically inappropriate. Instead, recurrence was evaluated through a qualitative, descriptive synthesis.

All statistical procedures were performed in the RStudio environment (R version 4.3.2), relying primarily on the meta and metafor libraries. When evaluating comparative endpoints between the two age cohorts, continuous data were synthesized using Weighted Mean Differences (WMDs), whereas categorical outcomes were assessed via Odds Ratios (ORs) derived from standard 2 × 2 contingency matrices. To quantify the between-study variance (t²) in these comparisons, the Restricted Maximum Likelihood (REML) method was applied.

For the synthesis of single-arm intraoperative and postoperative prevalence rates, we utilized the DerSimonian-Laird (DL) approach. Furthermore, to prevent extreme incidence rates (values nearing 0 or 1) from disproportionately skewing the assigned weights, variance was stabilized by applying the Freeman-Tukey double arcsine (PFT) transformation.

A random-effects and common-effects framework were utilized for all statistical evaluations according to the level of heterogeneity. Inter-study variance was quantified via Higgins’ I² index and Cochran’s Q test, with heterogeneity levels categorized sequentially as low (0–30%), moderate (30–50%), substantial (50–75%), and considerable (75–100%). The threshold for statistical significance was uniformly established at *p* < 0.05. Finally, to address clinical heterogeneity across varying hemorrhoidal disease categories, disease degree subgroup evaluations were carried out to compare the two surgical treatments across different levels of disease severity. Additionally, to account for the methodological variance between randomized and non-randomized evidence, sensitivity analyses were integrated to separately evaluate the pooled effect estimates of Randomized Controlled Trials against those of non-randomized prospective and observational cohorts. This stratification was necessary to ensure that potential selection bias and confounding by indication did not disproportionately skew the comparative findings. Furthermore, an assessment of cohort independence was conducted by cross-referencing the recruiting institutions and precise recruitment periods of all included studies. While the majority of cohorts were confirmed to represent entirely independent patient populations, a partial 10-month recruitment overlap was identified between two trials conducted at the same institution, Eskandaros et al. and Elhefny et al. [Supplementary File 2] [3, 8]. To eliminate the risk of double-counting overlapping patients, an additional sensitivity analysis was performed by excluding one of these studies from the pooled analyses to verify that this overlap did not artificially skew our overall findings.

### Data extraction

The retrieval of literature from the selected electronic databases was executed independently by two investigators (DC and NT), who subsequently recorded their findings within Microsoft Excel spreadsheets. The datasets primarily captured prevalence metrics and raw event counts. Following this independent extraction phase, the two authors cross-examined their respective databases, addressing any inconsistencies via mutual agreement. Notably, this entire extraction procedure was performed manually, without the integration of any automated extraction software.

### Risk of bias assessment

To evaluate the methodological quality and inherent risk of bias within the included literature, assessment tools were explicitly matched to the study design of each cohort. For the five Randomized Controlled Trials, the Cochrane Risk of Bias 2 (RoB 2) tool was utilized [9]. In contrast, for the five non-randomized prospective and observational cohorts, the Newcastle-Ottawa Scale (NOS) was applied [10]. This critical appraisal was carried out independently by two researchers (DC and NT). Under both frameworks, every specific domain was scored individually, culminating in a cumulative risk categorization (e.g., low risk, some concerns, or high risk for RoB 2; and high, moderate, or low for NOS). Any diverging evaluations between the reviewers were settled through detailed deliberation [Supplementary File 3] [Supplementary File 4]. Furthermore, potential publication bias was qualitatively evaluated through the visual inspection of funnel plots [Supplementary File 5].

## Results

**Fig. 1:**
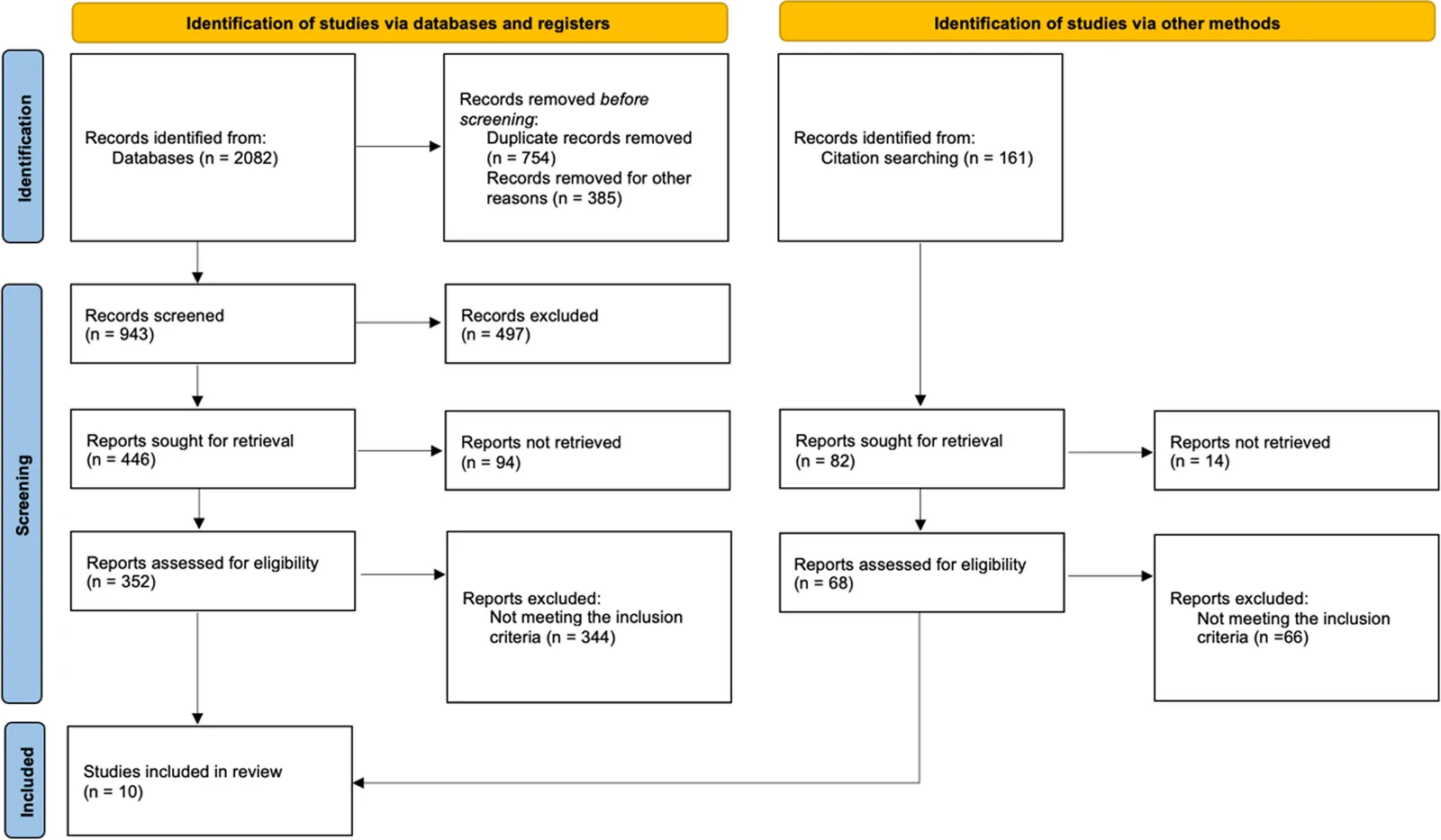
PRISMA flowchart

Our initial database queries, supplemented by manual citation tracking, yielded a combined total of 2,243 potential records (2,082 identified via databases and 161 via citation searching). Following the removal of duplicate entries and records excluded for other reasons prior to screening, the remaining records underwent initial screening. Unrelated reports were subsequently excluded, and the remaining reports were sought for retrieval. After excluding reports that could not be retrieved, the pool was narrowed to 420 manuscripts (352 from databases and 68 from other sources) for comprehensive full-text evaluation. Ultimately, 10 articles satisfied all necessary conditions and were synthesized within this review (Fig. 1). Furthermore, a detailed breakdown of the specific laser hemorrhoidoplasty techniques, including laser parameters, wavelengths, energy applications, and any adjunctive procedures utilized in each included study, is provided in Supplementary File 6.

A detailed breakdown of the baseline characteristics for these selected cohorts is provided below (Table 1).

**Table 1:** Study characteristics

Studies	Year	Hemorrhoids Grade	Patients with SH*	Patients with LH†	Study Design	Risk of Bias	Follow-up period	Recurrence^	Loss to follow up
Eskandaros et al. [8]	2020	3	40	40	RCT‡	Moderate Risk	1 year	Reported within 1 year	Not reported
Elhefny et al. [3]	2022	2, 3	30	30	RCT‡	Moderate Risk	1 year	Reported after 1 year	Not reported
Kaushal et al. [11]	2020	2, 3	25	25	Prospective Non-Randomized Comparative	Moderate Risk	Minimum: 3 months Maximum: 11 months	Reported after 1 week	Not reported
Bhanarkar et al. [4]	2025	2, 3	29	29	RCT‡	Moderate Risk	3 months	Up to 3 months	Not reported
Viswanath et al. [12]	2022	2, 3	36	36	Prospective Non-Randomized Comparative	Low Risk	6 months	Reported at 6 months	0
Sabry et al. [13]	2023	3	60	60	RCT‡	Moderate Risk	2 years	Reported after 2 years	12
Sinha et al. [5]	2025	3, 4	50	50	RCT‡	Moderate Risk	3 months	Not reported	Not reported
Goyal et al. [6]	2024	2, 3, 4	50	50	Prospective Non-Randomized Comparative	High Risk	3 weeks	Not reported	Not reported
Hung et al. [7]	2024	2,3,4	115	115	Prospective Non-Randomized Comparative	Low Risk	1 year	Reported at 5 months	Not reported
Hung et al. [14]	2020	2,3,4	200	204	Propensity-Score-Matched Prospective	Moderate Risk	Minimum: 4 months Maximum: 38 months	Reported within the follow up period	Not reported

*SH stapled hemorrhoidopexy

†*LH* laser hemorrhoidoplasty

^Definitions of recurrence were not provided by each study

‡ *RCT* randomized controlled trial

### Blood loss

**Fig. 2:**
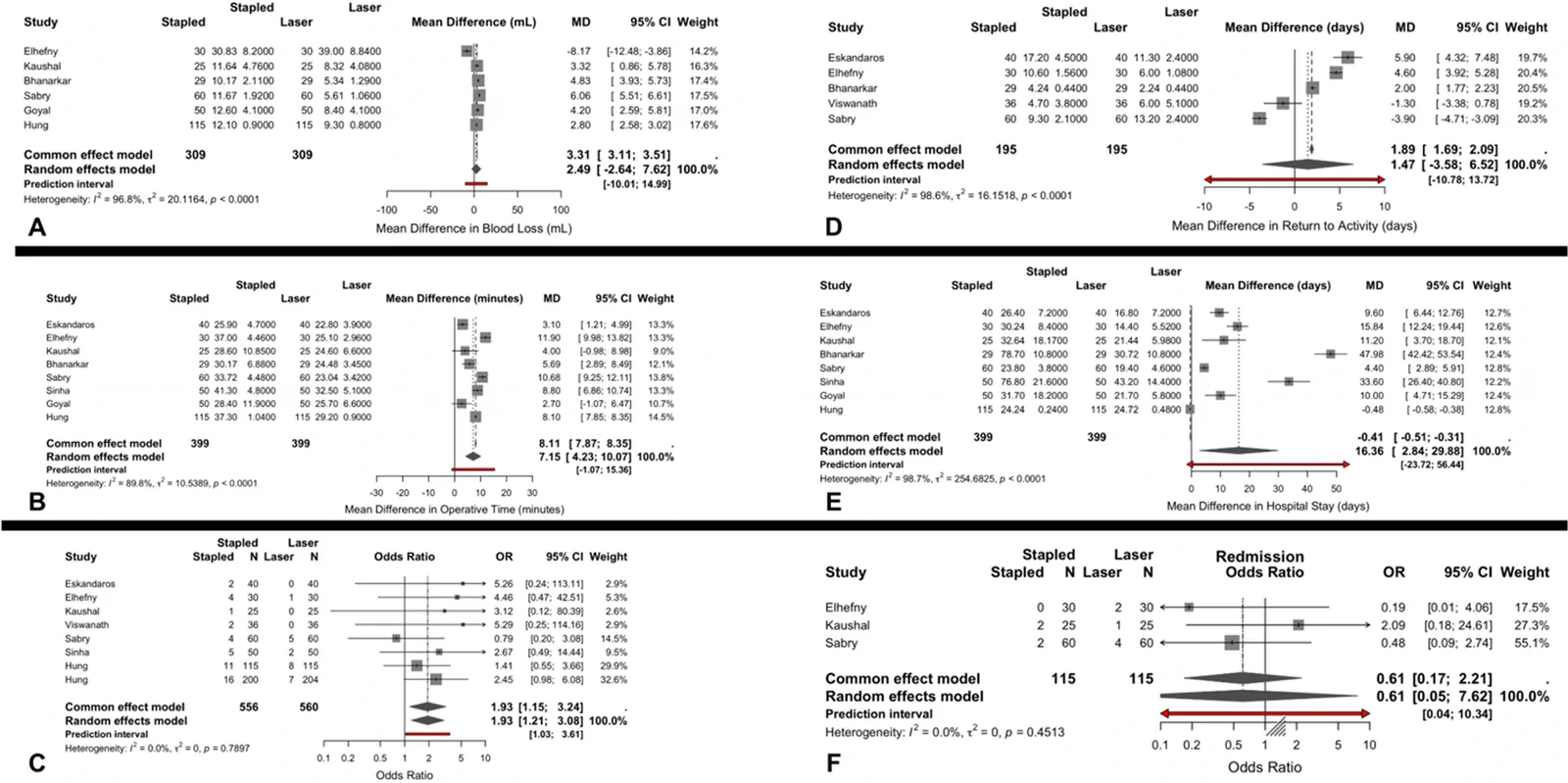
Comparing (**a**) blood loss, (**b**) total operative time, (**c**) urine retention, (**d**) return to normal activity, (**e**) hospital stay, and (**f**) readmission between laser hemorrhoidoplasty and stapled hemorrhoidopexy

The mean estimated blood loss during stapled hemorrhoidopexy was 13.9 ml and 11.7 ml in the laser group. Notably, the mean difference between the two was not found to be significant (MD = 2.49, 95% CI: -2.63; 7.62) (Fig. 2). Notably, blood loss mean difference did not vary significantly between the hemorrhoid types.

### Total operative time

Regarding the total operative time, the mean estimated time of stapled method was 33.0 min and 25.9 min for the laser group. The MD between the two values was found to be significant, meaning that the stapled approach required more time than the laser (MD = 7.14, 95% CI: 4.22; 10.06) (Fig. 2). Operative time mean difference did not vary according to the hemorrhoid type.

### Urine retention

The mean estimated prevalence of postoperative urine retention was 7.7% for the stapled approach and 3.0% for the laser cohort. The effect size for urine retention between the stapled and laser approach was OR = 1.92, 95% CI: 1.14; 3.24. The difference between the two groups was statistically significant (Fig. 2). Urine retention mean difference did not vary between hemorrhoid types.

### Return to normal activity

The estimated pooled outcome for the stapled method was 9.2 days and 7.7 in the laser group. The overall effect size between the two patient cohorts was not found to be significant, as patients with different approaches had similar return time frames (MD = 1.46, 95% CI: -3.58; 6.51) (Fig. 2). Subgroup analysis was not feasible.

### Postoperative hospital stay

The estimated mean length of stay was 40.1 h for the stapled approach and 23.7 h for the laser cohort. This variable was found to be significant between the stapled and laser approach, with MD = 16.3, 95% CI: 2.83; 29.87 (Fig. 2). Subgroup analysis based on the type of hemorrhoids did not show a significant difference.

### Readmission

The estimated prevalence of readmission was 2.6% for the stapled approach and 5.9% for the laser treatment. The effect size was not found to be significantly different between the two groups (OR = 0.60, 95% CI: 0.16; 2.21) (Fig. 2). Subgroup analysis was not feasible.

### Recurrence

In studies assessing early to mid-term follow-up (up to 6 months), recurrence rates observed to be higher following stapled hemorrhoidopexy. Specifically, Kaushal et al. (1 week), Bhanarkar et al. (up to 3 months), Viswanath et al. (6 months), and Hung et al. 2024 (5 months) reported SH recurrence rates ranging from 1.7% to 6.9%, compared to 0% to 3.4% for the LH groups. Two studies (Sinha et al. and Goyal et al.) did not report on recurrence during their brief follow-up periods.

Conversely, in studies with long-term follow-up (1 to 2 years), reported recurrence rates appeared higher in the laser hemorrhoidoplasty cohorts. At the 1-year mark, Eskandaros et al. reported a 10% recurrence for LH versus 7.5% for SH, while Elhefny et al. noted a 23.3% LH recurrence compared to 3.3% for SH. This pattern was also noted at 2 years, with Sabry et al. observing an 11.7% recurrence rate for LH versus just 1.7% for SH. Hung et al. 2020 was the sole cohort to report zero recurrences in both arms across its variable follow-up period. The detailed recurrence data across all studies can be seen in supplementary file 7.

### VAS pain 12h

**Fig. 3:**
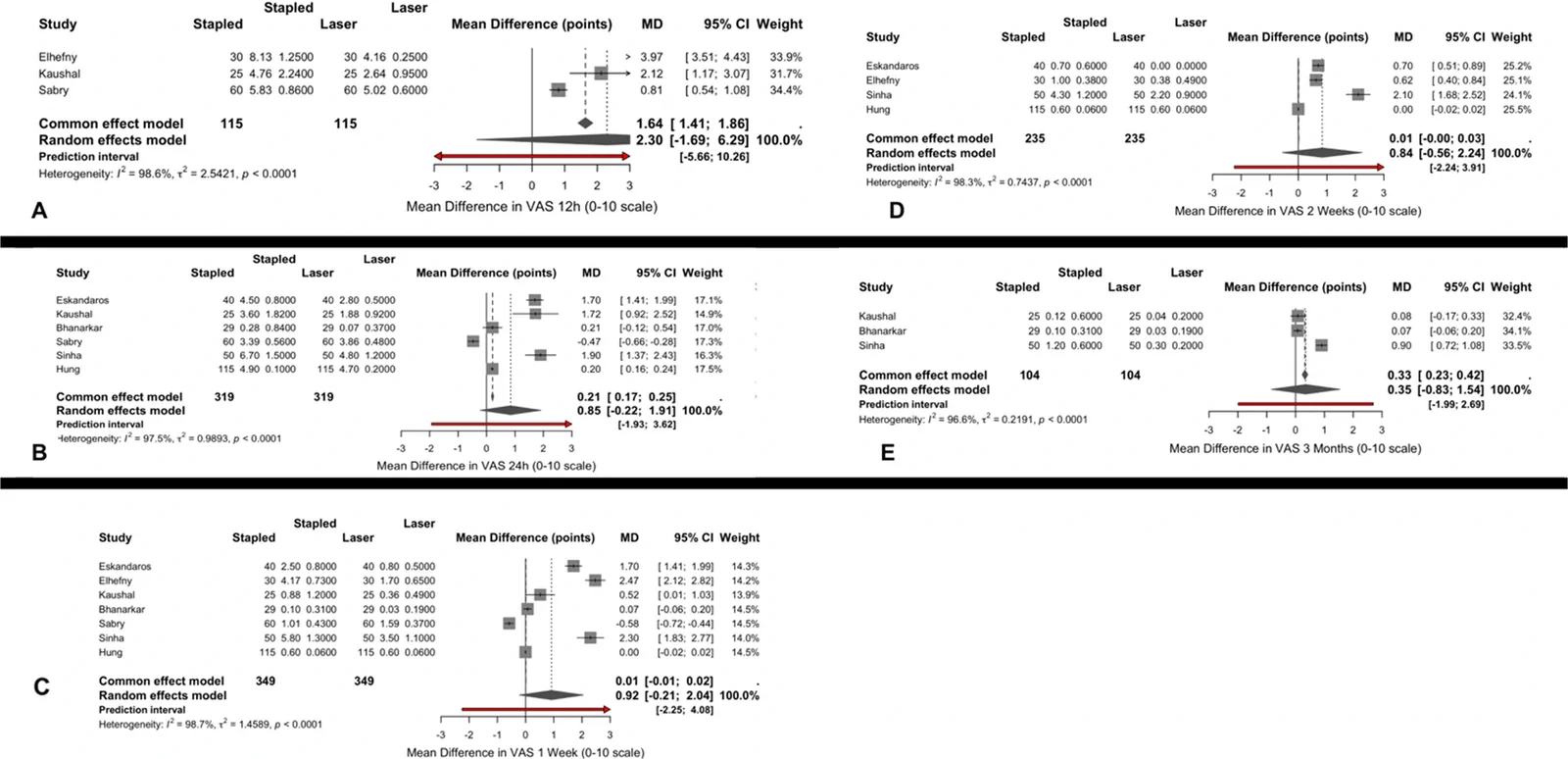
Comparing (**a**) VAS pain at 12 hours, (**b**) VAS pain at 24 hours, (**c**) VAS pain at 1 week, (**d**) VAS pain at 2 weeks, (**e**) VAS pain at 3 months between laser hemorrhoidoplasty and stapled hemorrhoidopexy

The estimated pooled mean of VAS pain at 12 h is 6.2 for the stapled cohort and 3.9 for the laser group. The effect size for VAS pain at 12 h was MD = 2.29, 95% CI: -1.69; 6.28. Notably, this outcome was not significantly different between the two groups (Fig. 3). Data did not allow us to compare the effect size between hemorrhoid types.

### VAS pain 24h

The estimated pooled mean of VAS pain at 24 h is 3.9 for the stapled method and 3.0 for the laser group. The effect size for VAS pain at 24 h was MD = 0.84, 95% CI: -0.22; 1.91. Notably, this outcome was not significantly different between the two groups (Fig. 3). The effect size MD did not vary between the hemorrhoid types.

### VAS pain 1 week

The estimated pooled mean of VAS pain at 1 week is 2.1 for the stapled method and 1.2 for the laser group. The effect size for VAS pain at 1 week was MD = 0.91, 95% CI: -0.21; 2.04. However, this outcome was not significantly different between the two groups (Fig. 3). The effect size MD did not vary between the hemorrhoid types.

### VAS pain 2 weeks

The estimated pooled mean of VAS pain at 2 weeks is 1.6 for the stapled method and 0.8 for the laser group. The effect size for VAS pain at 2 weeks was MD = 0.83, 95% CI: -0.56; 2.23. Notably, this outcome was not significantly different between the two groups (Fig. 3). The effect size MD did not vary between the hemorrhoid types.

### VAS pain 3 months

The estimated pooled mean of VAS pain at 3 months is 0.5 for the stapled cohort and 0.12 for the laser group. The effect size for VAS pain at 3 months was MD = 0.35, 95% CI: -0.83;1.53. However, this outcome was not significantly different between the two groups (Fig. 3). Subgroup analyses showed that the laser technique presented with a significant advantage over the stapled approach in the more severe cases, leaving patients with less postoperative pain at the 3-month mark.

### Bleeding

**Fig. 4:**
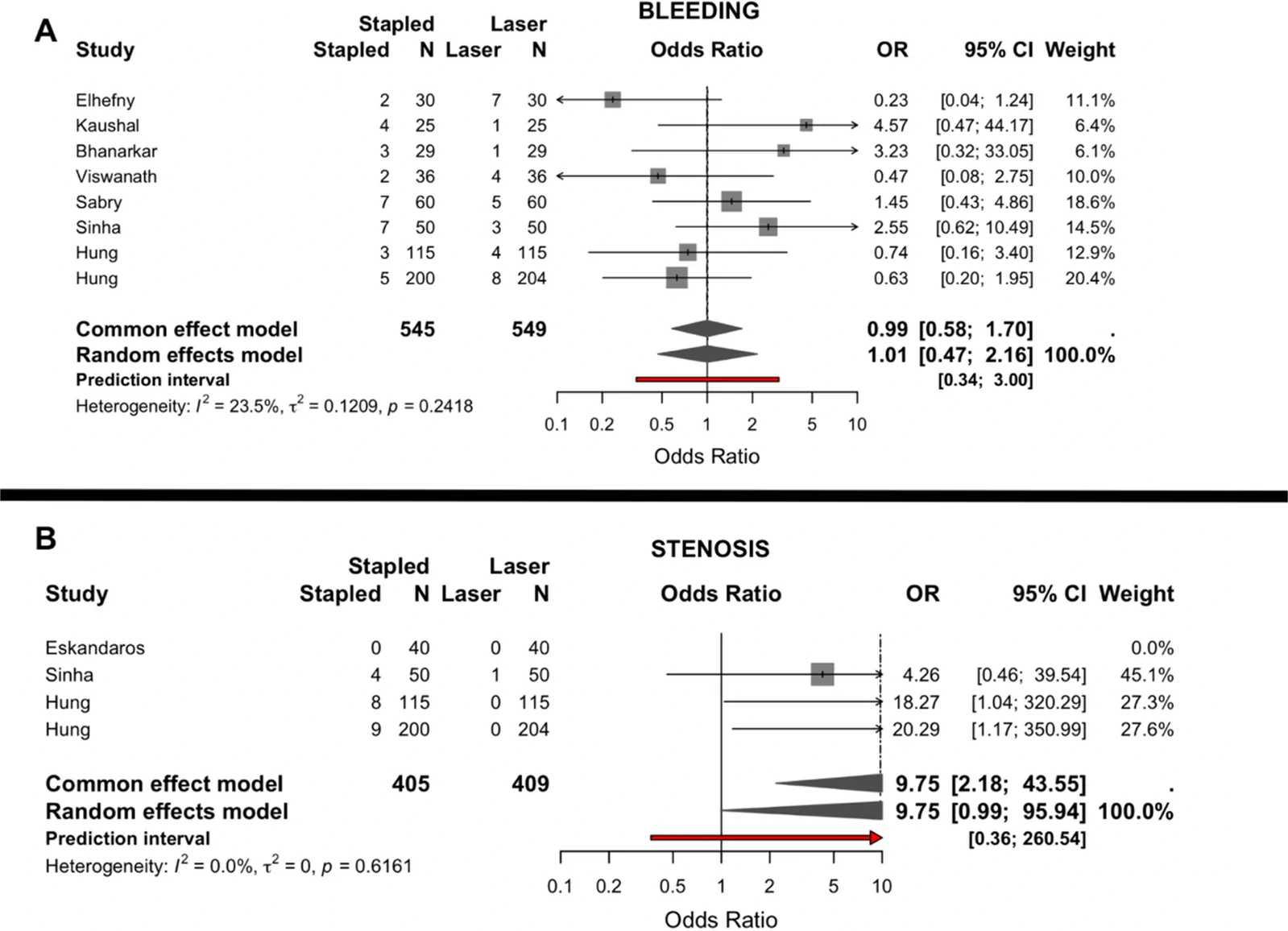
Comparing (**a**) bleeding and (**b**) stenosis complication rates between laser hemorrhoidoplasty and stapled hemorrhoidopexy

The estimated prevalence for bleeding complication was 7.0% in the stapled cohort and 6.2% in the laser group. The effect size of postoperative bleeding was OR = 1.00; 95% CI: 0.46; 2.15. This outcome, however, was not statistically different between the two groups (Fig. 4). The effect size OR did not vary between the hemorrhoid types.

### Stenosis

The estimated prevalence of stenosis was 4.5% for the stapled approach and 0% for the laser treatment. The effect size was found to be significantly different between the two groups (OR = 9.74, 95% CI: 2.18; 43.54) (Fig. 4). Subgroup analysis was not feasible.

A summary of the findings can be seen in Table 2.

**Table 2:** Main findings of the comparison

Parameters	Effect Size	Pooled outcome in stapled hemorrhoidopexy	Pooled outcome in laser hemorrhoidoplasty
Blood Loss (MD)	2.49 ml	13.9 ml	11.7 ml
Operative Time (MD)	7.14 min	33.0 min	25.9 min
Length of Stay (MD)	16.3 h	40.1 h	23.7 h
Urine retention (OR)	1.92	7.7%	3.0%
Return to normal activity (MD)	1.46 days	9.2 days	7.7 days
Readmission (OR)	0.60	2.6%	5.9%
Recurrence	Descriptive synthesis	Descriptive synthesis	Descriptive synthesis
VAS pain 12 h (MD)	2.29	6.2	3.9
VAS pain 24 h (MD)	0.84	3.9	3.0
VAS pain 1w (MD)	0.91	2.1	1.2
VAS pain 2w (MD)	0.83	1.6	0.8
VAS pain 3 m (MD)	0.35	0.5	0.12
Bleeding (OR)	1.0	7.0%	6.2%
Stenosis (OR)	9.74	4.5%	0.0%

Age cutoff subgroup analysis findings are in Supplementary File 8.

### Sensitivity analysis

Sensitivity analyses were conducted for each outcome using two distinct approaches. First, a leave-one-out analysis was performed where the study with the greatest weight was removed, and the effect size was recalculated. All outcomes did not change significance nor direction after the removal of the highest weighted study, indicating robust results concerning individual study influence.

Second, to directly address the methodological variance between randomized and non-randomized evidence, a dedicated sensitivity analysis was conducted by recalculating the pooled effect sizes after excluding the Randomized Controlled Trials. Across the majority of evaluated endpoints, the exclusion of RCTs did not alter the significance or direction of the results. However, two notable exceptions emerged: operative time and hospital stay, both of which shifted from statistically significant to non-significant when relying exclusively on observational data. Consequently, the original pooled findings for operative time and hospital stay are rendered less robust, and their clinical interpretation requires caution, as the overall statistical significance is highly sensitive to the underlying study design.

Third, to account for the identified partial recruitment overlap between two trials conducted at the same institution, an additional sensitivity analysis was performed by excluding one of the overlapping cohorts (Elhefny et al.). The recalculation of the affected pooled effect sizes demonstrated that all variables remained consistent, with no changes in statistical significance or direction. This confirms that the partial temporal overlap did not artificially skew our overall findings.

Visual inspection of funnel plots did not indicate any significant publication bias. Despite this, the fact that the small number of included studies did not allow us to conduct statistical tests for publications bias (e.g. Egger’s test), makes its evaluation not robust.

## Discussion

Various treatment options exist for the management of hemorrhoidal disease. However, because the condition presents with a wide spectrum of symptoms and severities, treatment approaches must be tailored to the degree of the disease. Third- and fourth-degree hemorrhoids typically require surgical intervention, with open hemorrhoidectomy widely considered the gold standard [5].

Nevertheless, alternative techniques such as laser hemorrhoidoplasty and stapled hemorrhoidopexy have been developed. While stapled hemorrhoidopexy is associated with less postoperative pain and shorter hospital stays, it also carries a risk of persistent complications, such as stenosis [5, 15, 16]. Conversely, studies indicate that the laser technique results in lower postoperative pain (VAS) scores and fewer overall complications [17, 18]. Therefore, the present study aims to directly compare the perioperative safety profiles and short-term recovery outcomes of these two alternative approaches.

Overall, our meta-analysis showed that as far as the majority of short-term patient outcomes are concerned, these two aforementioned techniques provide very similar results. Specifically, blood loss, return to normal activity, readmission, VAS pain (reported at 12 h, 24 h, 1 week, 2 weeks, and 3 months), and bleeding, were all comparable between the two. This result suggests that both treatment options can provide a comparable perioperative profile to manage hemorrhoids, without deteriorating the postoperative course of the patients (e.g. complications and pain). It is important to note that the absence of statistically significant differences in these metrics does not establish true clinical equivalence or non-inferiority. Nevertheless, it is important to distinguish between these immediate perioperative outcomes and actual long-term therapeutic efficacy. Because precise efficacy variables, like patient-reported outcomes and disease-specific functional outcomes, were insufficiently assessed across the primary literature, the true comparative efficacy of these procedures remains uncertain. The fact that the laser approach can present with similar safety and recovery metrics to the stapled approach has been supported by previous studies, which suggested that the improved tissue preservation and the controlled coagulation helped in the smoother postoperative course [5, 19].

The only significant differences that were observed were the operative time, hospital stay, and the postoperative complications, i.e. urinary retention and stenosis. The former was significantly longer in the stapled technique, while the laser counterpart completed the procedure in about 7 min less. Despite the statistical significance of this outcome, the clinical importance of such difference is questionable as it is unlikely to provide any meaningful advantage or alter workflow in a real-world surgical setting. As far as the hospital stay is concerned, the faster recovery is one of the main advantages of this approach, that has been reported in previous studies. This observation can be attributed to decreased complications and less/comparable postoperative pain [4, 5]. This makes laser hemorrhoidoplasty a great candidate for day-care procedures, while decreasing hospital and patient expenses [5]. However, reported differences in length of stay must be interpreted cautiously, as this metric is strongly influenced by institutional discharge policies, local healthcare protocols, and surgeon preference, rather than reflecting purely physiological recovery. Furthermore, it is important to note that the statistical significance of both operative time and hospital stay was lost during our sensitivity analysis when Randomized Controlled Trials were excluded. This discrepancy highlights how selection bias and confounding by indication, which are inherent to non-randomized, observational cohorts, can influence these specific perioperative metrics. Consequently, the pooled findings for operative time and hospital stay are less robust, and their clinical interpretation requires caution, as they are sensitive to the underlying study design. Regarding postoperative stenosis, despite the great effect size, which indicates a strong correlation between stapled hemorrhoidopexy and this complication, the majority of the data came from the same institution. Thus, this observation could be a result from a combination of factors, such as surgeon experience or hospital-related factors, and thus its interpretation needs caution. Finally, in terms of urinary retention, this complication is regarded as one of the most common postoperative complications following stapled hemorrhoidopexy [20]. Precisely, the reason and the pathophysiology behind such observation have not yet fully been explained but rather it is caused by a multifactorial process. Pelvic floor guarding due to pain, autonomic neural reflex inhibition, bladder overdistention, and the effects of perioperative opioids and intravenous fluids all contribute to the development of such complication. Additionally, the reflex sympathetic activation from postoperative pain, as well as the pelvic nerve irritation convey a role in the dysfunction of the detrusor muscle and in the increased urethral sphincter tone. Both of these changes can worsen urinary retention [20, 21].

Crucially, our evaluation of recurrence highlights the importance of analyzing outcomes by distinct follow-up intervals rather than pooling them into a single estimate. Because the follow-up periods across the primary literature were highly heterogeneous, calculating a pooled overall recurrence rate is methodologically unsound. Instead, our qualitative synthesis suggested that laser hemorrhoidoplasty appears to offer a lower risk of early recurrence (reported within 6 months), whereas stapled hemorrhoidopexy showed a potential trend toward better results at the 1- and 2-year marks. However, given the heterogeneous follow-up and undefined recurrence criteria, this observation should be considered purely hypothesis-generating rather than evidence of a true temporal difference in efficacy. While this qualitative interpretation precludes a definitive statistical consensus, it identifies a potential area that warrants targeted investigation in future long-term randomized trials.

Furthermore, the interpretation of the pooled results and subsequent subgroup analyses requires profound caution due to substantial clinical heterogeneity across hemorrhoidal grades. In clinical practice, stapled hemorrhoidopexy is typically reserved for more advanced, prolapsing disease, whereas laser hemorrhoidoplasty is frequently utilized for less advanced cases (grades II and III) [22, 23]. The primary literature included in this review exhibited considerable variation in disease severity, with most studies pooling outcomes across multiple grades (e.g., mixing grades II and III, or grades III and IV) rather than reporting isolated data for grade II, III, or IV disease independently. Consequently, our subgroup analyses could only classify studies based on whether they broadly included grade IV hemorrhoids, which inadequately resolves the underlying clinical heterogeneity. Because grade-specific data could not be analyzed independently, residual confounding by disease severity remains likely. Even though the fact that metrics like blood loss, operative time, urine retention, VAS pain (24 h, 1 week, 2 weeks), hospital stay, and bleeding did not significantly change across subgroups could suggest that the laser technique remains a comparable perioperative alternative to the stapled method, and the significantly lower VAS pain at 3 months in cohorts including grade IV hemorrhoids might even imply a small potential advantage for the laser approach in terms of postoperative morbidity, these findings must be interpreted with extreme caution. They could simply reflect the preoperative disease severity of the included patients. Conclusions regarding clinical equivalence, non-inferiority, or specific morbidity advantages cannot be definitively drawn when treatment allocation in the primary observational data was heavily influenced by this baseline severity.

Despite the potential advantages of the laser technique, including its minimally invasive nature, its increased costs due to specialized devices and fiber optics must also be considered [5]. Ultimately, further research is required to directly compare these techniques using isolated, grade-specific stratifications to eliminate confounding by indication and establish true comparative efficacy.

### Anatomical association

While the data generated by our meta-analysis focuses on comparing short-term and perioperative outcomes based on broad clinical grading, a comprehensive discussion must also acknowledge broader narrative considerations from the existing literature. Relying solely on clinical grading can provide an incomplete picture, as the ultimate choice of intervention should also be guided by the patient’s specific surgical anatomy. Previous studies have shown that most patients present with multi-quadrant involvement (~ 88%), while only a small minority actually has single-quadrant disease (~ 12%) [24]. Furthermore, hemorrhoidal disease often exhibits a varied clinical presentation, with individuals frequently developing at least two primary hemorrhoids of differing severity. This heterogeneity necessitates a multi-modal treatment approach over a single treatment choice, like laser ablation [24]. Thus, although anatomical variables were not directly evaluated in our pooled data, external literature suggests that the selection of surgical treatment should go beyond simply assessing the hemorrhoidal grade; a personalized approach should also account for the number and anatomical location of the piles [25].

## Strengths

The methodological strength of this meta-analysis is primarily rooted in our unbiased, dual-reviewer strategy for screening literature and extracting data. We ensured the robustness of our synthesized findings by strictly utilizing the Newcastle-Ottawa Scale for quality appraisal, alongside extensive calculations of prevalence rates and comparative effect sizes. Because we exclusively utilized open-source applications and standardized analytical models, our entire workflow is highly reproducible. Furthermore, the integrity of our results was rigorously supported through thorough sensitivity evaluations that evaluated the robustness of the findings. The inclusion of grade-stratified subgroup assessments provided more robustness to our findings. Finally, to account for the clinical and methodological variability inherent in observational surgical literature, random-effects models were utilized where appropriate to ensure the statistical soundness of the pooled data.

## Limitations

This systematic review and meta-analysis is subject to certain limitations. Notably, the majority of the included studies were observational in design. The limited number of randomized controlled trials means that the pooled data could be susceptible to selection bias, as patient allocation to the stapled or laser approach was not randomized and was likely influenced by surgeon preference and patient characteristics. Indeed, this methodological vulnerability was confirmed during our sensitivity analysis; when Randomized Controlled Trials were excluded, the previously significant differences in operative time and hospital stay were lost. This highlights that these specific perioperative metrics are highly sensitive to the underlying study design and were likely skewed.

Furthermore, our data synthesis is subject to clinical heterogeneity, as the primary studies pooled varying proportions of individual patient-level data. It was unfeasible to conduct separate, clean subgroup analyses or sensitivity checks for specific patient and preoperative variables (e.g. symptoms, age). Additionally, some degree of procedural heterogeneity was observed within the laser cohorts. Although we adopted the standardized term ‘laser hemorrhoidoplasty’ to encompass these interventions, the primary literature utilized varying laser systems and energy delivery protocols. Some studies also incorporated adjunctive surgical steps tailored to the patient’s specific anatomy. While these represent standard clinical adaptations, such technical variations could potentially influence perioperative outcomes like operative time or postoperative pain and grouping them under a single analysis is an inherent limitation of surgical meta-analyses that should be acknowledged. Crucially, there is a near-total absence of standardized patient-reported outcome measures and disease-specific functional tracking across the included literature. The primary cohorts did not systematically report on the sustained resolution of hemorrhoidal symptoms (such as prolapse, soiling, or pruritus). Because endpoints like postoperative pain scores and bleeding rates primarily reflect procedural morbidity rather than functional cure, this lack of data is a major limitation. Consequently, our findings are strictly limited to perioperative safety and short-term recovery metrics, precluding robust conclusions regarding comparative long-term therapeutic efficacy. Additionally, our evaluation of recurrence is inherently constrained by extreme heterogeneity in follow-up durations, making any definitive robust conclusion impossible. Furthermore, the inability of all included studies to provide a clear, standardized clinical definition of what constituted a recurrence is also a limitation, necessitating caution during interpretation of results. Similarly, the completeness of patient follow-up was poorly documented across the literature, with the exception of one study. This lack of reporting could introduce potential survivorship bias. Moreover, the limited number of eligible studies (< 10) precluded the performance of meta-regression analyses to explore additional variables. This small sample size also restricted our ability to perform formal statistical assessments for publication bias, such as Egger’s test or definitive visual assessment of funnel plots. Notably, the pooling of multiple hemorrhoidal grades within the primary literature prevented us from conducting isolated, grade-specific analyses. From a stricter proctological perspective, stapled hemorrhoidopexy and laser hemorrhoidoplasty have traditionally distinct clinical indications; however, the primary comparative studies in this meta-analysis reflect real-world practice where both procedures are utilized across overlapping disease stages (grades II through IV). Because surgical treatment allocation in these settings is influenced by this baseline disease severity, residual confounding by disease severity remains highly likely. This imbalance severely limits our ability to draw robust conclusions regarding the specific comparative advantages of these procedures across different stages of the disease. Similarly, the findings of the current subgroup analysis have limited value in determining the true advantages of each surgical approach in different hemorrhoidal grades. Ultimately, further research incorporating validated patient-reported outcomes, standardized protocols and stratified disease grades is needed to properly evaluate the clinical utility and true efficacy of these two surgical methods.

## Conclusion

This meta-analysis demonstrates that laser hemorrhoidoplasty and stapled hemorrhoidopexy showed no statistically significant differences across several reported short-term outcomes, including postoperative pain, bleeding, and time to return to normal activities. While the laser approach was associated with shorter operative times, reduced hospital stays, and lower rates of urinary retention and stenosis in the available studies, these findings must be interpreted with caution. Residual confounding by baseline disease severity and variations in underlying study design heavily influence these metrics, precluding definitive conclusions of superiority. Furthermore, it should be remembered that the lack of statistically significant differences in short-term metrics does not establish equivalence or non-inferiority between the procedures. Importantly, the true comparative therapeutic efficacy of these two techniques remains uncertain. Because the current literature lacks systematic reporting on validated patient-reported outcome measures, sustained symptom resolution, reintervention rates, and standardized recurrence measurements, this analysis permits conclusions regarding perioperative safety and recovery, but not robust conclusions regarding comparative long-term success. Apart from the direct comparison between the two methods, surgical anatomy of each individual patient should be taken into consideration, since the heterogenous nature of hemorrhoidal disease necessitates a multi-modal treatment approach, that combines more than one technique.

## Supplementary Information

Below is the link to the electronic supplementary material.

Supplementary Material 1 (DOCX 268 KB)

Supplementary Material 2 (DOCX 15.0 KB)

Supplementary Material 3 (DOCX 14.5 KB)

Supplementary Material 4 (DOCX 15.0 KB)

Supplementary Material 5 (PDF 73.9 KB)

Supplementary Material 6 (DOCX 17.8 KB)

Supplementary Material 7 (DOCX 15.2 KB)

Supplementary Material 8 (DOCX 14.9 KB)

## Data Availability

No datasets were generated or analysed during the current study.

## Declarations

### Use of AI

No AI was used for this manuscript.

### Disclaimer

No part of this manuscript is copied or published elsewhere in whole or in part.
