# Supplementary material for: Laser hemorrhoidoplasty vs. stapled hemorrhoidopexy: a meta-analysis of clinical outcomes with considerations in surgical anatomy
Source: Langenbecks Arch Surg. 2026 Nov 12;411(1):314. doi: 10.1007/s00423-026-04346-2 (PMC13759556; doi:10.1007/s00423-026-04346-2)
Supplement: Supplementary file 1 [file 423_2026_4346_MOESM5_ESM.pdf]

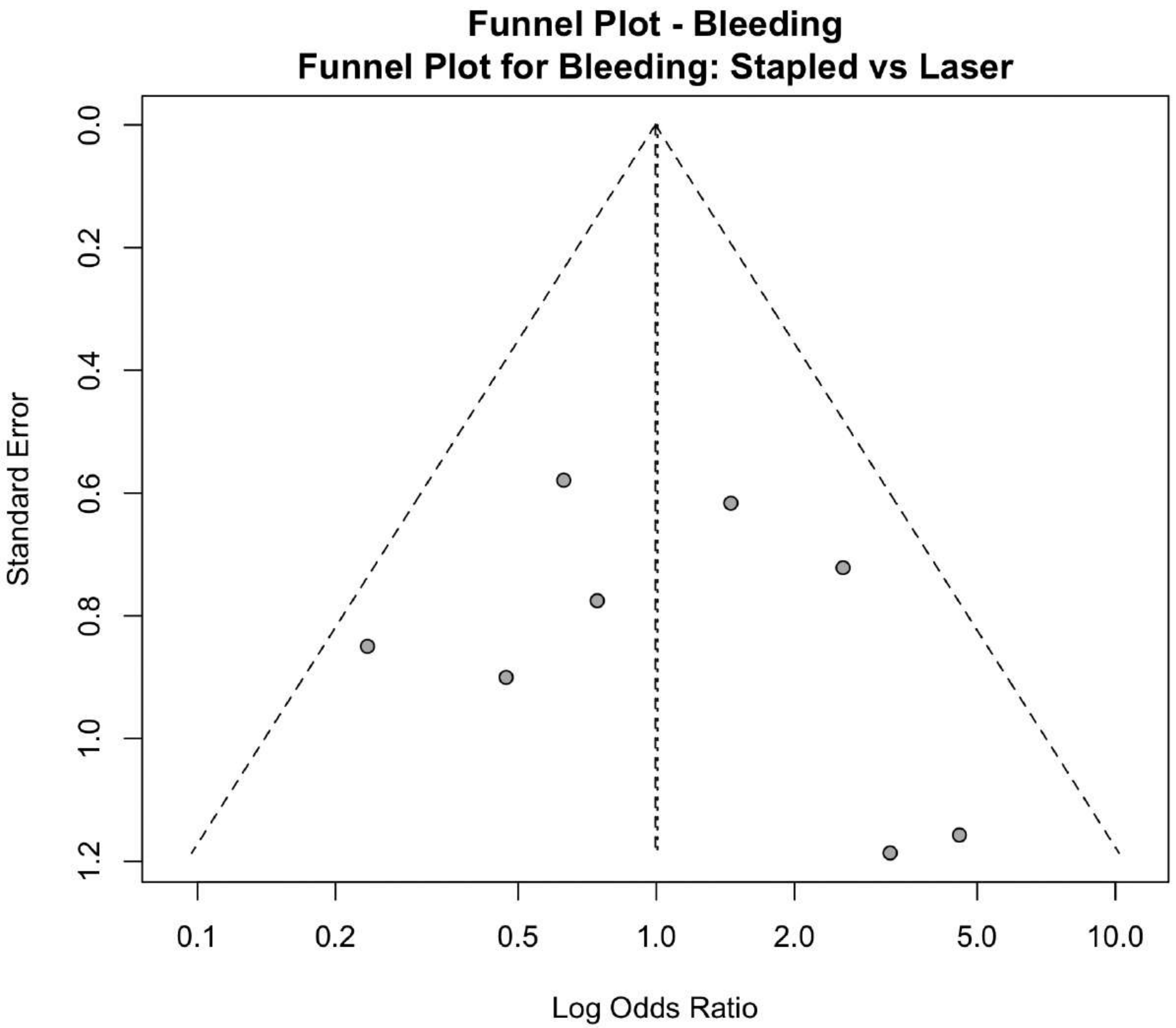
Funnel Plot - Bleeding
Funnel Plot for Bleeding: Stapled vs Laser
Standard Error
0.0
0.2
0.4
0.6
0.8
1.0
1.2
0.1
0.2
0.5
1.0
2.0
5.0
10.0
Log Odds Ratio

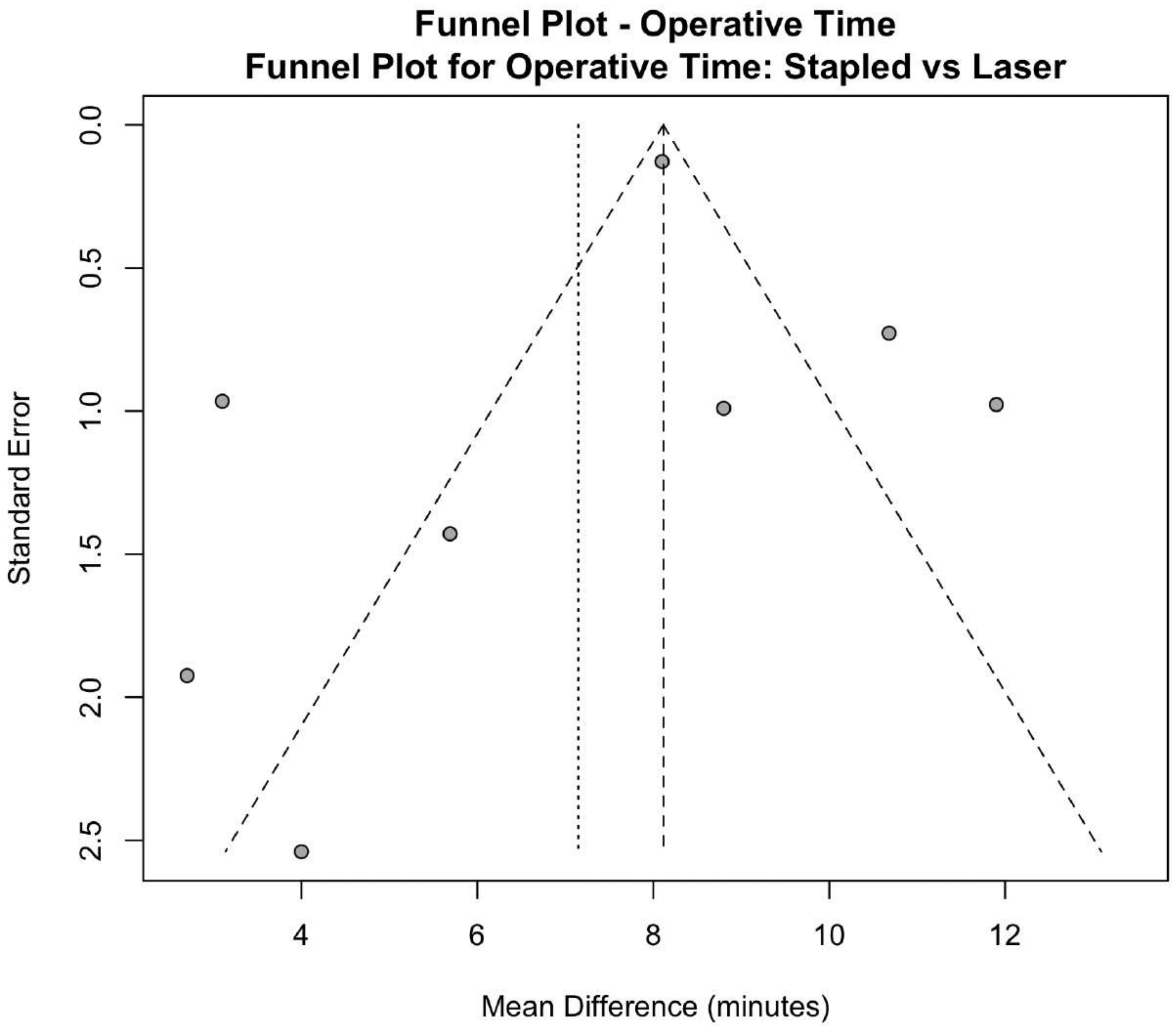
Funnel Plot - Operative Time
Funnel Plot for Operative Time: Stapled vs Laser
Standard Error
0.0
0.5
1.0
1.5
2.0
2.5
4
6
8
10
12
Mean Difference (minutes)
