# Supplementary material for: Laser hemorrhoidoplasty vs. stapled hemorrhoidopexy: a meta-analysis of clinical outcomes with considerations in surgical anatomy
Source: Langenbecks Arch Surg. 2026 Nov 12;411(1):314. doi: 10.1007/s00423-026-04346-2 (PMC13759556; doi:10.1007/s00423-026-04346-2)
Supplement: Supplementary file 3 [file 423_2026_4346_MOESM2_ESM.docx]

| Study | Institution | Time period |
| --- | --- | --- |
| Eskandaros et al. | Ain Shams  University Hospitals | April 2017 - October 2019 |
| Elhefny et al. | Ain Shams University Hospitals | January 2019 - January 2020 |
| Kaushal et al. | Artermis Hospitals | April 2018 - September 2019 |
| Bhanarkar et al. | Government Medical College and Hospital Nagpur | August 2022-July 2024 |
| Viswanath et al. | ASTER Malabar Institute of Medical Sciences | June 2019 - May 2020 |
| Sabry et al. | Ain  Shams University Hospitals | March 2020 - March 2022 |
| Sinha et al. | Indira Gandhi  Institute of Medical Sciences | - |
| Goyal et al. | Index Medical College Hospital & Research Centre | - |
| Hung et al. | Binh Dan Hospital | March 2022 - March 2023 |
| Hung et al. | Binh Dan Hospital | September 2016 - September 2019 |

Supplementary Table 1: Data collection characteristics from each study
