# Supplementary material for: Laser hemorrhoidoplasty vs. stapled hemorrhoidopexy: a meta-analysis of clinical outcomes with considerations in surgical anatomy
Source: Langenbecks Arch Surg. 2026 Nov 12;411(1):314. doi: 10.1007/s00423-026-04346-2 (PMC13759556; doi:10.1007/s00423-026-04346-2)
Supplement: Supplementary file 4 [file 423_2026_4346_MOESM3_ESM.docx]

| Studies | Selection | Comparability | Outcome | Overall |
| --- | --- | --- | --- | --- |
| Kaushal et al. | 4/4 | 0/2 | 2/3 | Fair |
| Viswanath et al. | 4/4 | 1/2 | 3/3 | Very Good |
| Goyal et al. | 3/4 | 0/2 | 1/3 | Poor |
| Hung et al. | 4/4 | 2/2 | 3/3 | Very Good |
| Hung et al. | 4/4 | 0/2 | 2/3 | Fair |

Supplementary Table 2: Risk of Bias Assessment based on the Newcastle-Ottawa Scale
