# Supplementary material for: Laser hemorrhoidoplasty vs. stapled hemorrhoidopexy: a meta-analysis of clinical outcomes with considerations in surgical anatomy
Source: Langenbecks Arch Surg. 2026 Nov 12;411(1):314. doi: 10.1007/s00423-026-04346-2 (PMC13759556; doi:10.1007/s00423-026-04346-2)
Supplement: Supplementary file 5 [file 423_2026_4346_MOESM4_ESM.docx]

| Studies | Eskandaros et al. | Elhefny et al. | Bhanarkar et al. | Sabry et al. | Sinha et al. |
| --- | --- | --- | --- | --- | --- |
| Domain 1: Randomization | Some concerns | Some concerns | High risk | Some concerns | Some concerns |
| Domain 2: Deviation from intended interventions | Some concerns | Some concerns | Some concerns | Some concerns | Some concerns |
| Domain 3: Missing outcome data | Low risk | Low risk | Low risk | Some concerns | Low risk |
| Domain 4: Measurement of outcome | High risk | High risk | High risk | High risk | High risk |
| Domain 5: Selection of reported result | Some concerns | Some concerns | Some concerns | Some concerns | Some concerns |
| Overall | Some concerns | Some concerns | Some concerns | Some concerns | Some concerns |

Supplementary Table 3: Risk of Bias Assessment based on the Cochrane RoB 2
