# Supplementary material for: Laser hemorrhoidoplasty vs. stapled hemorrhoidopexy: a meta-analysis of clinical outcomes with considerations in surgical anatomy
Source: Langenbecks Arch Surg. 2026 Nov 12;411(1):314. doi: 10.1007/s00423-026-04346-2 (PMC13759556; doi:10.1007/s00423-026-04346-2)
Supplement: Supplementary file 6 [file 423_2026_4346_MOESM6_ESM.docx]

Supplementary Table 5: Laser hemorrhoidoplasty techniques used in each study

| Studies | Laser system/Wavelength | Energy Application | Columns treated | Adjunctive procedures |
| --- | --- | --- | --- | --- |
| Eskandaros et al. | Ceralas diode laser Biolitic system / 1470 nm | Pulsed fashion: 3 seconds on, 0.5 seconds off. | Base of each hemorrhoid | Ice finger applied intra-anally for 0.5–1 min for cooling. |
| Elhefny et al. | Ceralas diode laser Biolitic system / 980 nm and 1470 nm | 10 W (980 nm) / 4 W (1470 nm). Pulses of 3 seconds on, 0.5 seconds off. | Base of each hemorrhoid | Excision of mucosal prolapse and skin tags using continuous wave mode. Ice finger applied internally for 0.5–1 min for cooling. |
| Kaushal et al. | Diode laser with bare optical fiber / 1470 nm | 13 W. Up to 6 pulses per pedicle (3 seconds on, 1.2 seconds off). | 3 positions (all hemorrhoids) | Mucopexy at 2, 4, 8, and 10 o'clock for significant prolapse. Excision of external hemorrhoids/redundant tissue. Gauze pressure applied for 30 seconds for cooling/edema. |
| Bhanarkar et al. | Lasotronix system / 1470 nm (bare tip 1000-micron fiber) | 15 Watts; 200–300 Joules per pile mass. Delivered in 5 pulses (1.2 seconds on, 0.6 seconds off) | Single pile masses | None reported |
| Viswanath et al. | Diode laser / 1470 nm | 8 W. 150–350 Joules per segment, delivered in pulsed fashion. | Base of each hemorrhoid | None reported |
| Sabry et al. | Biolitec diode laser / 980 nm (using a 1000-micron optical fiber) | 3 W. Pulse mode: 5 laser pulses, 1.2 seconds on, 0.6 seconds off | Not specified | None reported |
| Sinha et al. | Diode laser (Lasotronix) / Wavelength not specified | Continuous pulse mode | Not specified | None reported |
| Goyal et al. | Diode laser / Wavelength not specified | Pointed laser probe inserted submucosally via a 2mm nick. | Not specified | Short distance mucopexy above the dentate line was added for patients with significant mucosal prolapse. |
| Hung et al. | Semiconductor laser generator (Biolitec) / 1470 nm | 3 W laser power. The laser fiber was inserted into the submucosa, advanced to the hemorrhoid cushion, and activated | Many piles | Suture ligation (degree 2) or mucopexy ligation (degree 3 or 4).  Additional resection of skin tags if necessary.  Cooling with a cool pack applied intra-operatively |
| Hung et al. | Semiconductor laser generator (Biolitec) / 1470 nm | 15 W laser power. Pulse mode: 3 seconds on, 1 second off. Energy distributed half under the mucosa and half in the hemorrhoidal segment | Every hemorrhoidal segment | Stitching applied on the dentate line 2 to 4 cm if hemorrhoids prolapsed more than 2 cm from the dentate line.  Ice applied to the treated hemorrhoids for 30 to 45 seconds after completing every segment.  Excision of skin tags under local anesthesia if required |
