# Supplementary material for: Laser hemorrhoidoplasty vs. stapled hemorrhoidopexy: a meta-analysis of clinical outcomes with considerations in surgical anatomy
Source: Langenbecks Arch Surg. 2026 Nov 12;411(1):314. doi: 10.1007/s00423-026-04346-2 (PMC13759556; doi:10.1007/s00423-026-04346-2)
Supplement: Supplementary file 7 [file 423_2026_4346_MOESM7_ESM.docx]

| Study names | Recurrence SH* | Recurrence LH† | Recurrence |
| --- | --- | --- | --- |
| Eskandaros et al. | 7.5% | 10% | Reported within 1 year |
| Elhefny et al. | 3.3% | 23.3% | Reported after 1 year |
| Kaushal et al. | 4% | 0% | Reported after 1 week |
| Bhanarkar et al. | 6.9% | 3.4% | Up to 3 months |
| Viswanath et al. | 2.8% | 0% | Reported at 6 months |
| Sabry et al. | 1.7% | 11.7% | Reported after 2 years |
| Sinha et al. | - | - | Not reported |
| Goyal et al. | - | - | Not reported |
| Hung et al. | 1.7% | 0% | Reported at 5 months |
| Hung et al. | 0% | 0% | Reported within the follow up period |

Supplementary Table 6: Recurrence data across all studies

†LH: Laser hemorrhoidoplasty

*SH: Stapled hemorrhoidopexy
