# Supplementary material for: Laser hemorrhoidoplasty vs. stapled hemorrhoidopexy: a meta-analysis of clinical outcomes with considerations in surgical anatomy
Source: Langenbecks Arch Surg. 2026 Nov 12;411(1):314. doi: 10.1007/s00423-026-04346-2 (PMC13759556; doi:10.1007/s00423-026-04346-2)
Supplement: Supplementary file 8 [file 423_2026_4346_MOESM8_ESM.docx]

| Parameters | Studies with grades II-IV | Studies with grades II-III | P-value |
| --- | --- | --- | --- |
| Blood loss (MD) (ml) | 3.26 | 1.77 | 0.6476 |
| Operative time (MD) (min) | 6.94 | 7.28 | 0.8941 |
| Urinary retention (OR) | 1.97 | 2.07 | 0.9130 |
| Time to normal activity (MD) (hours) | - | 1.46 | - |
| Hospital stay (MD) (hours) | 14.12 | 17.72 | 0.7763 |
| Readmission (OR) | - | 0.60 | - |
| VAS pain 12 h (MD) | - | 2.29 | - |
| VAS pain 24h (MD) | 1.02 | 0.76 | 0.7919 |
| VAS pain 1 week (MD) | 1.13 | 0.83 | 0.8102 |
| VAS pain 2 weeks (MD) | 1.03 | 0.66 | 0.7231 |
| VAS pain 3 months (MD) | 0.90 | 0.07 | <0.0001* |
| Bleeding (OR) | 1.00 | 1.02 | 0.9734 |
| Stenosis (OR) | 9.74 | - | - |

Supplementary Table 7: Subgroup analysis results
